# Resveratrol Ameliorates Renal Damage, Increases Expression of Heme Oxygenase-1, and Has Anti-Complement, Anti-Oxidative, and Anti-Apoptotic Effects in a Murine Model of Membranous Nephropathy

**DOI:** 10.1371/journal.pone.0125726

**Published:** 2015-05-08

**Authors:** Chia-Chao Wu, Yen-Sung Huang, Jin-Shuen Chen, Ching-Feng Huang, Sui-Lung Su, Kuo-Cheng Lu, Yuh-Feng Lin, Pauling Chu, Shih-Hua Lin, Huey-Kang Sytwu

**Affiliations:** 1 Division of Nephrology, Department of Medicine, Tri-Service General Hospital, National Defense Medical Center, Taipei, Taiwan; 2 Graduate Institute of Microbiology and Immunology, National Defense Medical Center, Taipei, Taiwan; 3 Institute of Biomedical Sciences, Academia Sinica, Taipei, Taiwan; 4 Department of Pediatrics, Tri-Service General Hospital, National Defense Medical Center, Taipei, Taiwan; 5 School of Public Health, National Defense Medical Center, Taipei, Taiwan; 6 Department of Medicine, Cardinal Tien Hospital, School of Medicine, Fu Jen Catholic University, New Taipei city, Taiwan; 7 Division of Nephrology, Department of Medicine, Shuang-Ho Hospital, Taipei Medical University, Taipei, Taiwan; University of South Carolina School of Medicine, UNITED STATES

## Abstract

**Background:**

Idiopathic membranous nephropathy (MN) is an autoimmune-mediated glomerulonephritis and a common cause of nephrotic syndrome in adults. There are limited available treatments for MN. We assessed the efficacy of resveratrol (RSV) therapy for treatment of MN in a murine model of this disease.

**Methods:**

Murine MN was experimentally induced by daily subcutaneous administration of cationic bovine serum albumin, with phosphate-buffered saline used in control mice. MN mice were untreated or given RSV. Disease severity and pathogenesis was assessed by determination of metabolic and histopathology profiles, lymphocyte subsets, immunoglobulin production, oxidative stress, apoptosis, and production of heme oxygenase-1 (HO1).

**Results:**

MN mice given RSV had significantly reduced proteinuria and a marked amelioration of glomerular lesions. RSV also significantly attenuated immunofluorescent staining of C3, although there were no changes of serum immunoglobulin levels or immunocomplex deposition in the kidneys. RSV treatment of MN mice also reduced the production of reactive oxygen species (ROS), reduced cell apoptosis, and upregulated heme oxygenase 1 (HO1). Inhibition of HO1 with tin protoporphyrin IX partially reversed the renoprotective effects of RSV. The HO1 induced by RSV maybe via Nrf2 signaling.

**Conclusion:**

Our results show that RSV increased the expression of HO1 and ameliorated the effects of membranous nephropathy in a mouse model due to its anti-complement, anti-oxidative, and anti-apoptotic effects. RSV appears to have potential as a treatment for MN.

## Introduction

Idiopathic membranous nephropathy (MN) is an autoimmune-mediated glomerulonephritis that is characterized by deposition of immunocomplex over the subepithelial space [1, 2]. These immune complex deposits and the subsequent responses, including complement activation, oxidative injury, inflammation, and apoptosis, appear to be central to the pathogenesis of MN [3–5]. MN is one of the most common causes of nephrotic syndrome in adults, and approximately 30–40% of patients with MN progress to renal impairment and ultimately to end-stage renal failure after 10–15 years. In recent decades, the treatment of MN with immunosuppressive drugs has improved patient outcomes. However, presently available therapies are not always effective and often have persistent adverse effects [6–8]. Therefore, there is uncertainty and disagreement about the most appropriate treatment for MN.

Resveratrol (*trans-*3,4,5-trihydroxy-*trans-*stilbene, RSV) is a polyphenolic phytoalexin that naturally occurs in many foods including grapes, red wine, and some berries [9]. There is recent attention on the potential therapeutic effects of RSV because it may partly explain the “French paradox”—low incidence of cardiovascular disease among the French due to red wine consumption [10, 11]. Previous research has documented that RSV has antioxidant [12, 13], anti-inflammatory [14, 15], estrogenic [16], antiplatelet [17], anticancer [18, 19], and cardioprotective properties [10, 11, 20]. Studies of the effects of RSV in various animal models indicated that it can effectively treat or prevent several diseases [21, 22]. However, no studies have yet examined the effects of RSV on autoimmune MN.

In this study, we investigated the therapeutic effects of RSV on MN and examined the mechanisms of this response in a murine model of this disease. In particular, we examined the effect of RSV on pathological injury to the glomeruli, oxidative stress, apoptosis, and activation of heme oxygenase 1 (HO1) in order to assess the feasibility of using RSV as a new therapy for MN.

## Materials and Methods

### Mice

All animal experiments were approved by the Animal Care and Use Committee of the National Defense Medical Center, Taipei, Taiwan (IACUC-15-043) and all experiments were conducted according to the National Institutes of Health Guidelines. BALB/c female mice (4–6 weeks old, >20 g body weight) were initially purchased from the National Laboratory Animal Center (Taipei, Taiwan) and were maintained under specific-pathogen-free conditions in the Laboratory Animal Center of the National Defense Medical Center.

### Experimental design

Initially, the mice were randomly assigned to an experimental group or a normal control group. The animals in both groups were immunized with 0.2 mg of cationic bovine serum albumin (cBSA) emulsified in an equal volume of complete Freund’s adjuvant [23, 24]. Two weeks later, the MN group received cBSA (13 mg/kg) intravenously three times per week (every other day) for six weeks, and the normal control (NC) group received pure saline on the same schedule. Mice in each group were then randomly assigned to two subgroups (6 mice each), which received daily subcutaneous injections of 30 mg/kg bodyweight of RSV (MN-RSV and NC-RSV groups) or vehicle (control 5% ethanol) (MN and NC groups) starting from the time of MN induction. RSV prepared as a stock solution of 30 mg/mL in 5% ethanol (injection volume was 1 mL/kg bodyweight). The same design was used for an HO1 inhibition experiment, in which tin (IV) protoporphyrin IX chloride (SnPP, a competitive inhibitor of HO1; Frontier Scientific, Logan, UT, USA) or PBS was administered. Homogeneous cBSA was prepared as previously described [25, 26]. Disease severity was assessed by serum and urine metabolic profiles and by renal histopathology.

### Serum and urine measurements

Urine samples were collected every week for protein measurement by a urine dipstick (Albustix, Bayer, Tarrytown, NY, USA). Grade 0–4 proteinuria was defined by concentrations of 0–30, 30–100, 100–300, 300–2000, and more than 2000 mg/dL, respectively. Blood samples were collected, microcentrifuged, and stored at -70°C until analysis. The concentrations of blood urea nitrogen (BUN), creatinine, albumin, and total cholesterol were determined with a Fuji DRI-CHEM 3030 (Fuji Photo Film Co., Ltd, Tokyo, Japan). The concentrations of BUN, Cr, albumin, and total cholesterol were determined using commercially available kits (Roche Diagnostics, Indianapolis, IN). Serum concentrations of anti-cBSA IgG1, and IgG2a were measured as described previously, and data are expressed as OD_450 nm_ [27]. All assays were performed in duplicate, according to the manufacturer’s instructions.

### Histology of renal tissues

The mice were anesthetized and their kidneys harvested after perfusion with PBS to remove blood from the tissues *via* the abdominal aorta. Formalin-fixed paraffin-embedded sections of the kidney tissues were cut and stained with hematoxylin and eosin (H&E) and silver stain. Frozen sections were air dried, fixed in acetone, and washed with PBS before incubation with fluorescein isothiocyanate (FITC)-conjugated goat anti-mouse immunoglobulin G (IgG), C3, C4, or C1q (Capple, Durham, NC, USA) for immunofluorescence analysis. Fluorescence was observed as described previously [24].

### Immunohistochemistry

The formaldehyde-fixed paraffin-embedded tissues were immunohistochemically stained. The endogenous peroxidase activity was quenched, and the sections were blocked with 1% (w/v) BSA in PBS for 1 h. The sections were then incubated with a 1:300 dilution of rabbit polyclonal anti-HO1 (Stressgen Biotechnologies, Victoria, BC, Canada) in PBS, followed by horseradish-peroxidase-conjugated goat anti-rabbit IgG antibody (Novus Biologicals, Littleton, CO, USA). The reaction products were visualized with a solution of 3-amino-9-ethylcarbazole (AEC; Dako, Carpinteria, CA, USA), and slides were counterstained with hematoxylin.

### Flow cytometry

All monoclonal antibodies were purchased from BD Biosciences (San Jose, CA, USA) or eBioscience (San Diego, CA, USA). Lymphocytes harvested from the spleens were stained with different marker-specific antibodies: allophycocyanin-conjugated anti-mouse CD4 (clone GK1.5), phycoerythrin-conjugated anti-mouse CD8a (clone 53–6.7), or FITC-conjugated anti-mouse CD19 (clone 1D3; BD Biosciences Pharmingen). The stained cells were analyzed with a FACSCalibur cell sorter using CellQuest software (Becton Dickinson, San Jose, CA, USA).

### Measurement of renal reactive oxygen species (ROS)

In situ superoxide anion production was determined by labeling with dihydroethidium (DHE; Molecular Probes, Eugene, OR, USA), as previously described [11]. Briefly, 15 μm-thick, frozen sections were incubated with 10 μmol/L DHE at 37°C for 30 min in a humidified chamber that was protected from light. The fluorescent images were quantified by counting the percentage of positive nuclei per kidney cross section [24].

### Terminal deoxynucleotidyl transferase-mediated nick end-labeling (TUNEL) assay

The TUNEL assay was used to measure apoptosis using the In Situ Cell Death Detection Kit (Roche Molecular Biochemicals, Mannheim, Germany). Briefly, kidney sections were fixed with 4% paraformaldehyde and washed with PBS. The cells were permeabilized with 0.1% Triton X-100 in a 0.1% sodium citrate solution, rinsed with PBS, and incubated for 1 h in the TUNEL reaction mixture (terminal deoxynucleotidyl transferase with FITC-conjugated dUTP). After washing in PBS, the slides were examined with a fluorescence photomicroscope (Olympus, Tokyo, Japan).

### Cell culture and siRNA transfection

The podocyte cell line E11 used had been previously described in detail[28]. E11 cells were maintained in RPMI-1640 supplemented with 10% fetal bovine serum (FBS), and penicillin or streptomycin at 33°C under a humidified atmosphere of 5% CO2. E11 cells at 50% confluence were thermo-switched from 33°C to 38°C for 14 days. Differentiated podocytes were transfected with ON-TARGET plus SMARTpool siRNA against Nrf2 or non-target control (GE Dharmacon, Lafayette, USA) for 72 h using Lipofectamine RNAiMAX (Invitrogen, Carlsbad, USA) according to the manufacturer’s instructions. Further experiments were conducted in triplicates using indicated concentrations of resveratrol at 6 h.

### RNA extraction and real-time quantitative PCR

Total RNA was extracted from the renal cortex with the TRIzol Reagent (Life Technologies, Gaithersburg, MD, USA), according to the manufacturer’s protocol. Total RNA (5 μg) was reverse transcribed with oligo(dT) primer using the SuperScript III Reverse Transcriptase kit (Life Technologies). Real-time polymerase chain reaction (PCR) analyses were performed using the SYBR Green Master Mix Kit (Bio-Rad) and the Opticon PCR thermal cycler (MJ Research, Waltham, MA, USA). The relative expression of each mRNA was normalized to that of *Gapdh*. All experiments were performed three times in duplicate. The forward primer/reverse primer probe sequences are gccaccaaggaggtacacat/gcttgttgcgctctatctcc for HO-1 and gccaaaagggtcatcatctc/ggccatccacagtcttct for GAPDH. The forward primer / reverse primer probe sequences are ttcgggaaagtgaaggtggg / gatgtgagggtgcctgaaca for AMPK1, tcggcaaagtgaagattggaga / tgccactttatggcctgtca for AMPK2, gtcccagcaggacatggatt / gctcatagtccttctgtcgct for Nrf2, ccgatcgcaatggaagaag / cgtatgagtctttctggccca for γ-actin.

### Immunoprecipitation and Western Blot Analysis

E11 cells were lysed directly in RIPA buffer (Thermo Scientific, Lithuania, USA) containing protease inhibitor (Sigma, St. Louis, USA). 20 μg of protein were loaded in each lane of 8~10% SDS—PAGE gels, transferred onto Immobilon-P Transfer Membrane (Millipore, Bedford, USA), probed with antibodies, and analyzed with Las-4000 imaging system (Fujifilm, Valhalla, USA). 200 ug of cell lysates were mixed with antibody and the immunocomplexes were collected on Dynabeads protein A (Life Technologies, Carlsbad, USA). Antibodies are indicated as following: anti-Nrf2, anti-AMPK1/2, anti-β-actin (Cell Signaling, Danvers, USA), control rabbit IgG (Santa Sruz, Dallas, USA) and anti-HO1 (ENZO Biotech, New York, USA).

### Chromatin Immunoprecipitation Quantitative PCR (ChIP-qPCR) Assay

ChIP was performed as previously described[28]. ChIP product was analyzed by quantitative real-time PCR using the Applied Biosystem 7500 Real-Time PCR System. Experiments were done in triplicate. A fraction (1%) of the sonicated chromatin was set aside as input control before antibody affinity manipulations. Percent input was calculated by 100 x 2^(Ct^adjusted Input^—Ct^IP^). Purified DNA was subjected to qPCR, using specific primers for mouse HO1 promoter: region 1 (-656 ~ -523), cctgggcagaacacttcaac and ttctgcttggtttgctgacac; region 2 (-483 ~ -350), agttccgggaaggtagactca and cagtgacaggaagacgggag; region 3 (-130 ~ -18), tgatttatccccttacaggcagg and gccagccctttaagtacgc; region 4 (-42 ~ +33), tgacccgcgtacttaaaggg and tatgcggaaactctggaggc.

### Statistical analysis

Data are expressed as means ± standard deviations. Statistical analysis with one-way ANOVA was performed for multiple comparisons, and the Bonferroni test was used to correct for between-group differences. Differences were considered significant if the *p*-value was less than 0.05. Statistical analyses were performed with SPSS/PC (SPSS Inc., Chicago, IL, USA).

## Results

### Effect of RSV on laboratory indicators of MN

**Fig 1:**
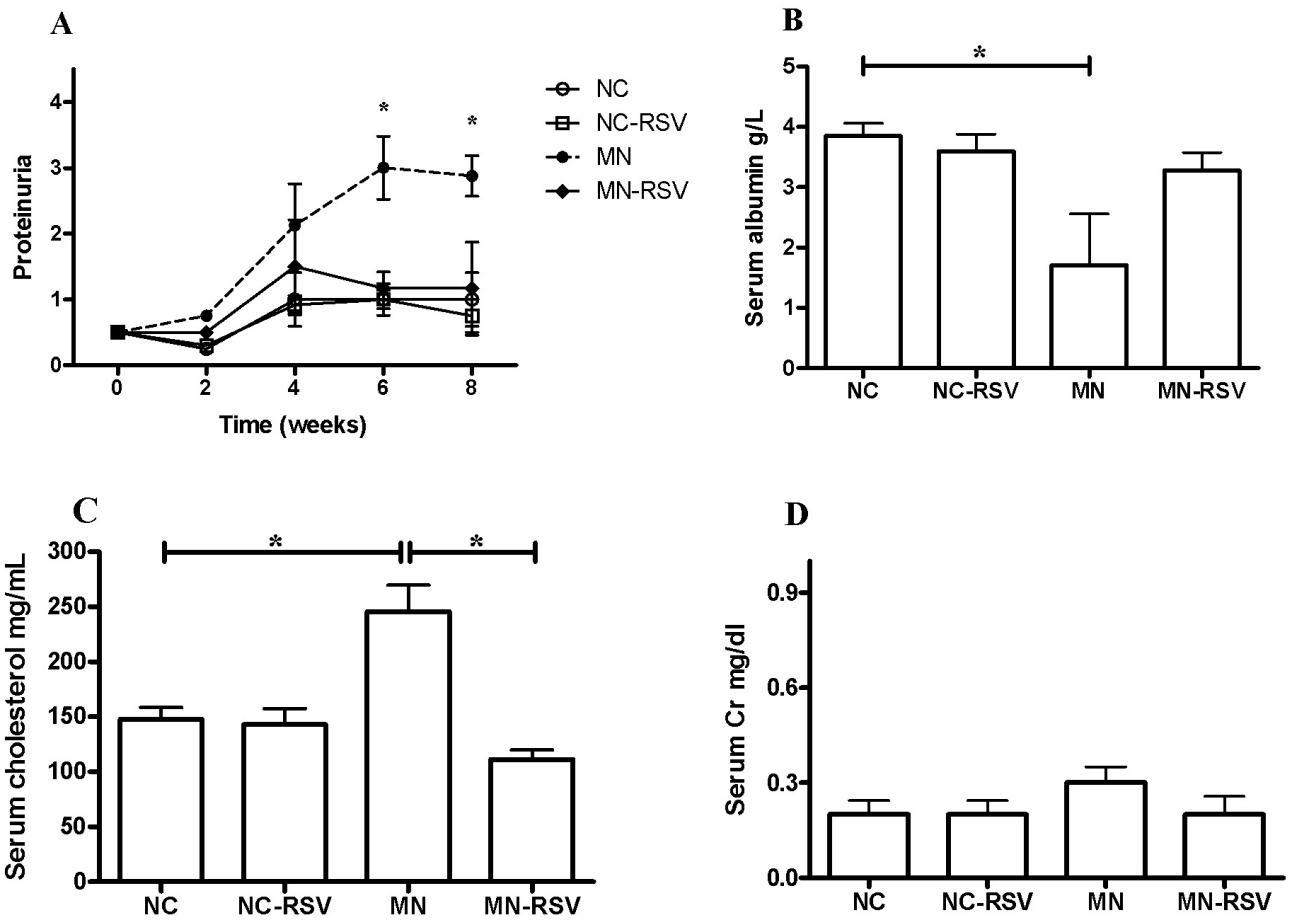
Effect of resveratrol on laboratory indicators of membranous nephropathy. (A) proteinuria, (B) serum albumin, (C) serum cholesterol, (D) serum creatinine. Abbreviations: NC, normal control; NC-RSV, NC with resveratrol; MN, membranous nephropathy; MN-RSV, MN with RSV; *, *p <* 0.05.

Mice with experimentally induced MN had pathologies characteristic of nephrotic syndromes, including proteinuria, hypoalbuminemia, and hypercholesterolemia; RSV treatment significantly attenuated these 3 pathological responses (Fig 1A–1C). Although the serum creatinine levels of the MN mice were elevated, there were no significant differences among the 4 groups (Fig 1D). The general characteristics of the NC- RSV group were also not significantly different from those of the NC group.

### Effect of RSV on renal histology

**Fig 2:**
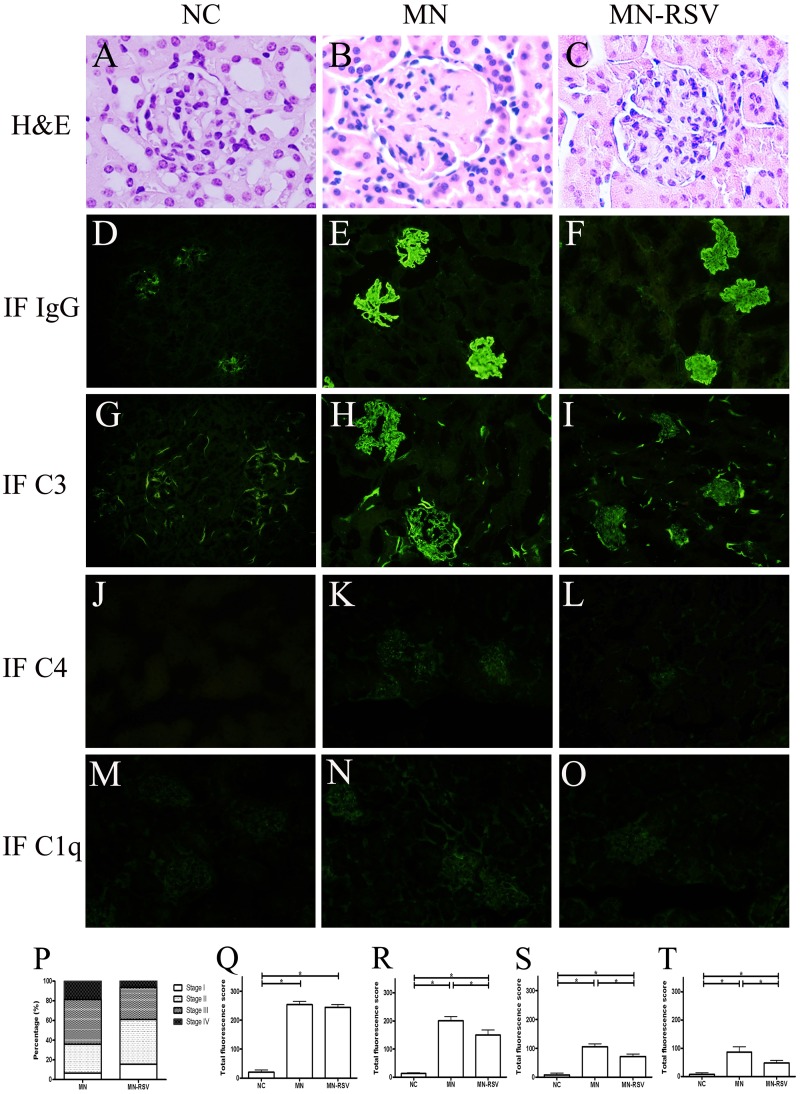
Effect of resveratrol on renal histology. Representative kidney sections from mice in the NC group (A, D, G, J, M), MN group (B, E, H, K, N), and MN-RSV group (C, F, I, L, O) following H&E staining (A–C) and immunofluorescence staining of IgG (D–F), C3 (G–I), C4 (J–L), and C1q (M–O). The quantifications of H&E staining (P) and immunofluorescence staining of IgG (Q), C3 (R), C4 (S), and C1q (T) were listed. All images are ×400. Abbreviations as in Fig 1. *, *p <* 0.05.

Histological analysis by H&E staining indicated that NC mice had normal renal tissues, MN mice had diffuse thickening of the glomerular basement membrane, and MN-RSV mice had less severe pathology than MN mice (Fig 2A–2C). MN mice also had greater immunofluorescence staining for IgG than NC mice, with a discrete beaded appearance along the glomerular capillary wall (Fig 2D–2F); the results were similar in the MN and MN-RSV mice, suggesting that RSV treatment did not change the deposition of immune complexes. Immunofluorescence staining for C3 indicated increased granular fluorescence along the glomerular capillary wall in MN mice, and that RSV treatment attenuated this response (Fig 2G–2I). The results for C4 (Fig 2J–2L) and C1q (Fig 2M–2O) were similar to those for C3, although the fluorescence signals were weaker. These findings indicated that RSV treatment affect the classical pathway for activation of the complement system and has anti-complementary activity. The histopathological and immunofluorescence features of the NC-RSV mice did not differ from those of NC mice (data not shown).

### Effect of RSV on lymphocyte subsets and immunoglobulin production

**Fig 3:**
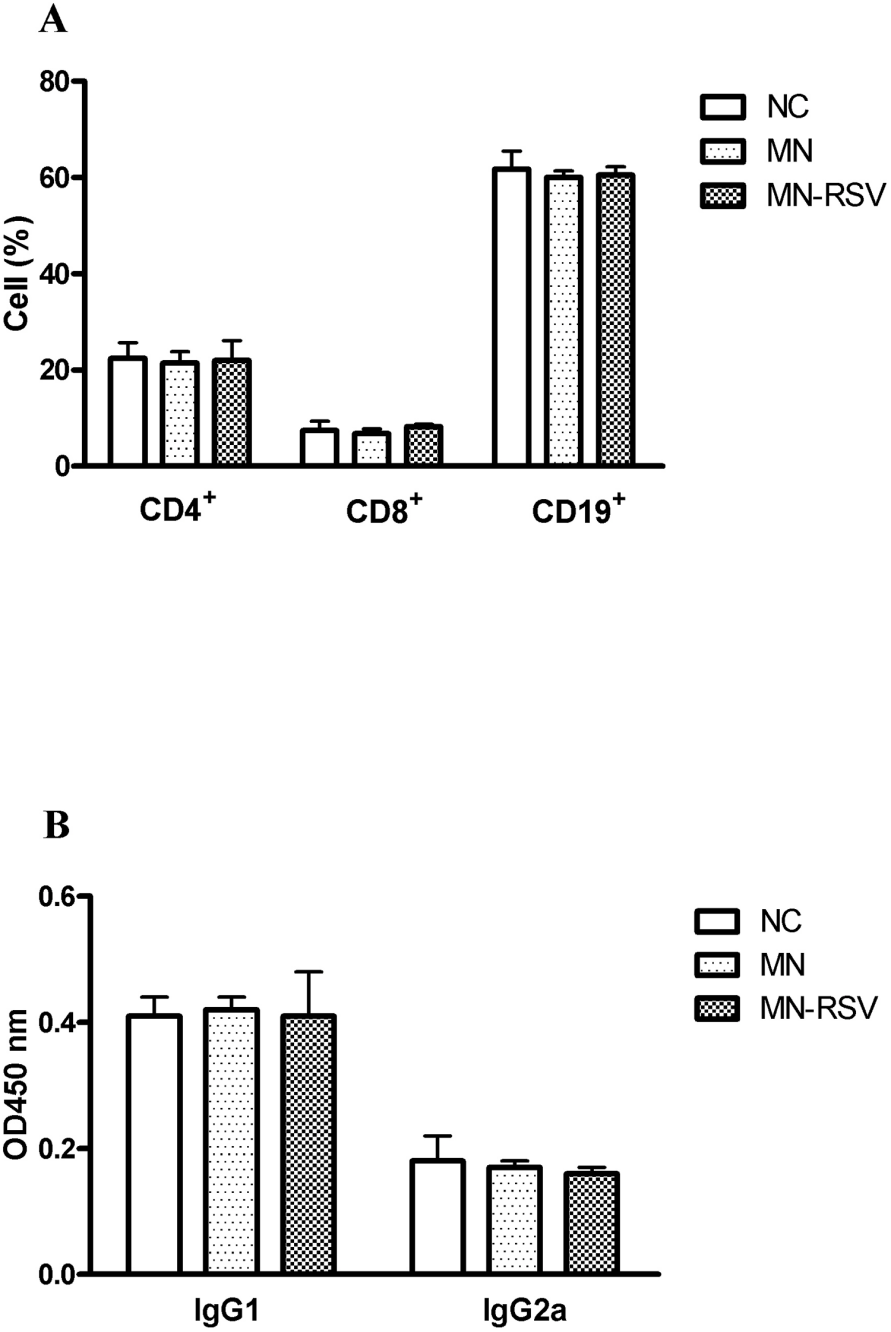
Effect of resveratrol on distribution of lymphocyte subsets and production of immunoglobulins. (A) Percentages of CD4^+^, CD8^+^, and CD19^+^ immune cells and (B) serum anti-cBSA immunoglobulins IgG1 and IgG2a. Each bar shows the mean ± standard deviation of 5 mice. Abbreviations as in Fig 1. *, *p <* 0.05.

Immune cells have important roles in the development and progression of MN. Thus, we tested the effect of MN and RSV treatment on the development of lymphocytes by analysis of splenic lymphocytes with flow cytometry. The results show no significant differences in the populations of CD4^+^ T cells, CD8^+^ T cells, or CD19^+^ B cells for mice in the NC, MN, and MN-RSV groups (Fig 3A). We also investigated the effect of MN and RSV treatment on the production of immunoglobulins. Again, there were no significant differences in the levels of IgG1 or IgG2a in the 3 groups (Fig 3B). These results demonstrate that MN and RSV treatment did not affect antibody production, and suggest that MN and RSV treatment may have a direct effect on anti-complement activity.

### Effect of RSV on ROS production and apoptosis

**Fig 4:**
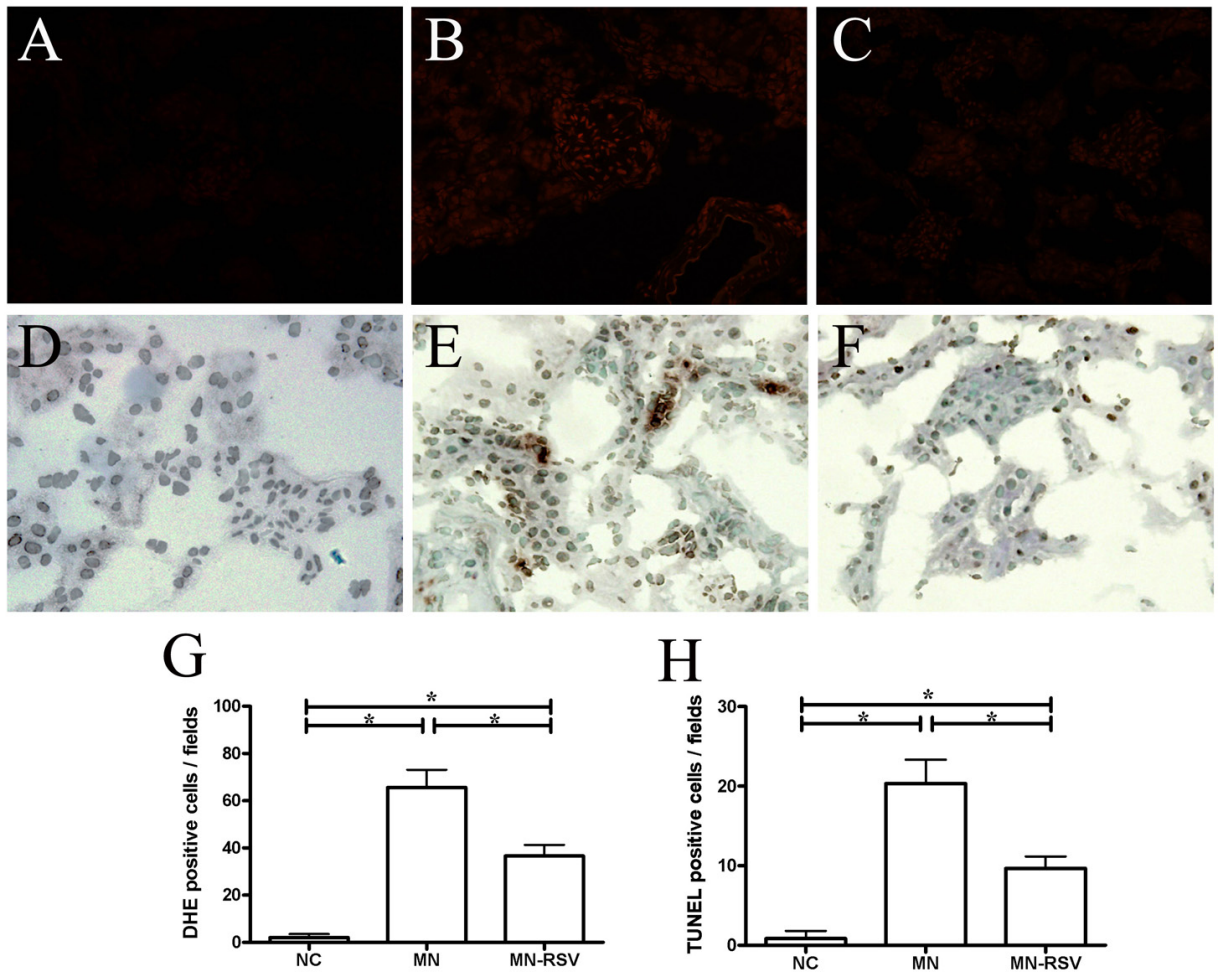
Effect of resveratrol on production of superoxide anion and kidney cell apoptosis. (A-C, G) Fluorescence micrographs of dihydroethidium (DHE)-positive cells in the kidneys of mice from the NC, MN, and MN-RSV groups (respectively) and their quantification. (D-F, H) Fluorescence micrographs of TUNEL-positive cells in the kidneys of mice from the NC, MN, and MN-RSV groups (respectively), and their quantification. All images are ×400. Abbreviations as in Fig 1. *, *p <* 0.05.

We also used the DHE assay to analyze the production of superoxide anion radical in renal tissues (Fig 4A–4C and 4G). The levels of DHE fluorescence were low in NC mice, significantly increased in MN mice, and attenuated in MN-RSV mice. Thus, MN increased the production of ROS in renal tissues and that RSV treatment partially reversed this effect.

RSV has known antiapoptotic effects, so we also investigated its effect on apoptosis in the renal tissues of MN mice (Fig 4D–4F and 4H). NC mice had virtually no TUNEL-positive cells (an index of cell apoptosis), MN mice had a large number of apoptotic cells in the glomeruli and surrounding tubules, and RSV treatment partially reversed this effect. Thus, the antiapoptotic effect of RSV may contribute to its therapeutic efficacy against MN.

### Role of HO1 in RSV treatment

**Fig 5:**
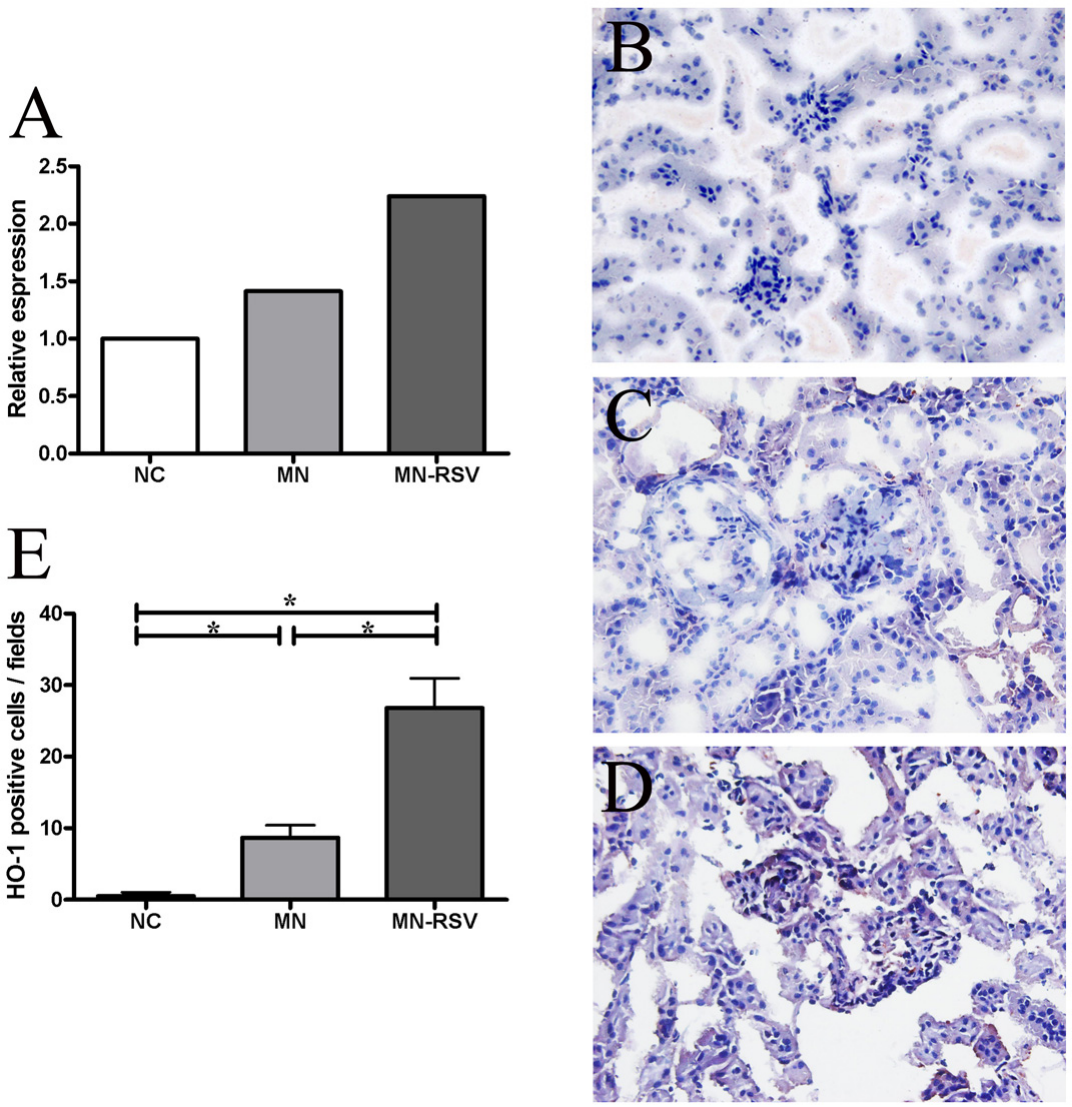
Effect of resveratrol on renal expression of heme oxygenase-1. (A) Expression of HO1 mRNA of mice in the NC, MN, and MN-RSV groups, (B-D, E) immunohistochemical staining of HO1 protein of mice in the NC, MN, and MN-RSV groups and their quantification. All images are ×400. Abbreviations as in Fig 1. *, *p <* 0.05.

Previously, we demonstrated that heme oxygenase 1 (HO1), the rate-limiting enzyme in the degradation of heme into carbon monoxide (CO), ferritin, and biliverdin, was highly expressed during the induction of MN, and that HO1 induction ameliorated MN due to its anti-oxidative, anti-apoptotic, and immunomodulatory effects [26]. A recent study reported that RSV may induce HO1 [29]. Thus, we used real-time PCR to examine the effect of MN and RSV treatment on expression of HO1 in the kidney (Fig 5A). The results show that NC mice had low expression, MN mice had ~1.5-fold higher expression, and MN-RSV mice had ~2.2-fold higher expression (Fig 5A). We also examined expression of HO1 protein in the kidneys of mice using immunohistochemistry (Fig 5B–5D and 5F). NC mice had very weak expression of HO1 in the glomeruli and tubules of the cortical sections, MN mice had slightly elevated expression, and MN-RSV mice had greatly increased expression in the glomeruli and renal tubules.

**Fig 6:**
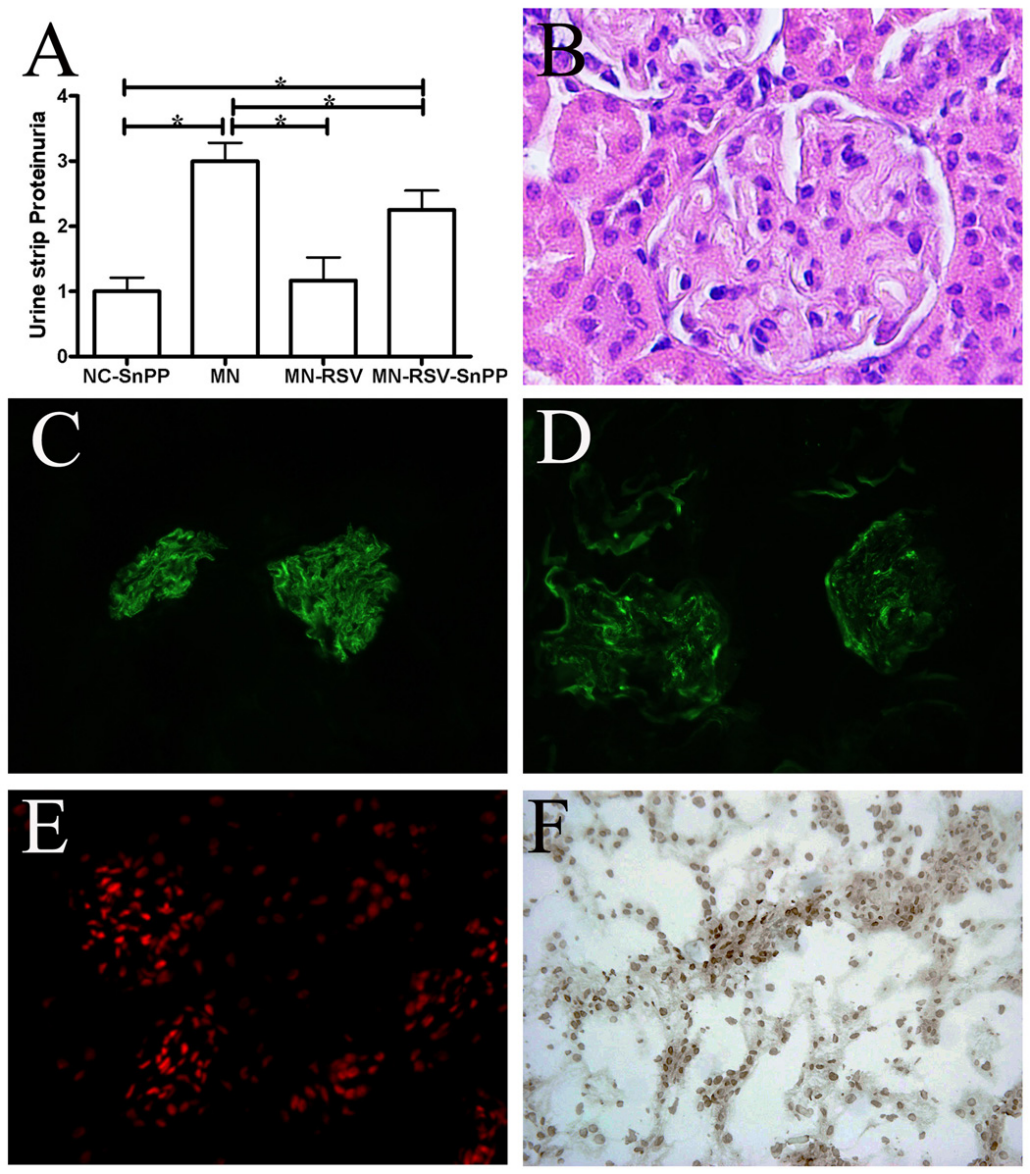
Effect of heme oxygenase-1 inhibition on the renoprotective effects of resveratrol. (A) Proteinuria in controls (MN and MN-RSV groups) and in mice given an HO1 competitive inhibitor (NC-SnPP and MN-RSV-SnPP groups). (B-F) Representative kidney sections of mice in the MN-RSV-SnPP group were subjected to H&E staining, IgG staining, C3 staining, DHE staining, and TUNNEL staining, respectively. All images are ×400. Abbreviations as in Fig 1. *, *p <* 0.05.

We further assessed the effect of HO1 inhibition during RSV treatment by administration of SnPP, a competitive inhibitor of HO1 (Fig 6). MN mice had greatly elevated proteinuria, MN-RSV-SnPP mice had somewhat reduced proteinuria, and MN-RSV mice had greatly attenuated proteinuria (Fig 6A). Compared with the renal histology of MN-RSV mice, MN-RSV-SnPP mice had more severe renal pathology (Fig 6B *cf*. Fig 2C), greater immunofluorescence staining for IgG (Fig 6C *cf*. Fig 2F) and C3 (Fig 6D *cf*. Fig 2I), stronger DHE fluorescence (Fig 6E *cf*. Fig 4C), and more TUNEL-positive nuclei (Fig 6F *cf*. Fig 4F). Thus, inhibition of HO1 by SnPP attenuated the RSV-mediated induction of HO1 activity and hindered the renoprotective effects of RSV during MN.

### RSV activates HO1 expression via stimulating Nrf2 binding activity in E11 podocytes

**Fig 7:**
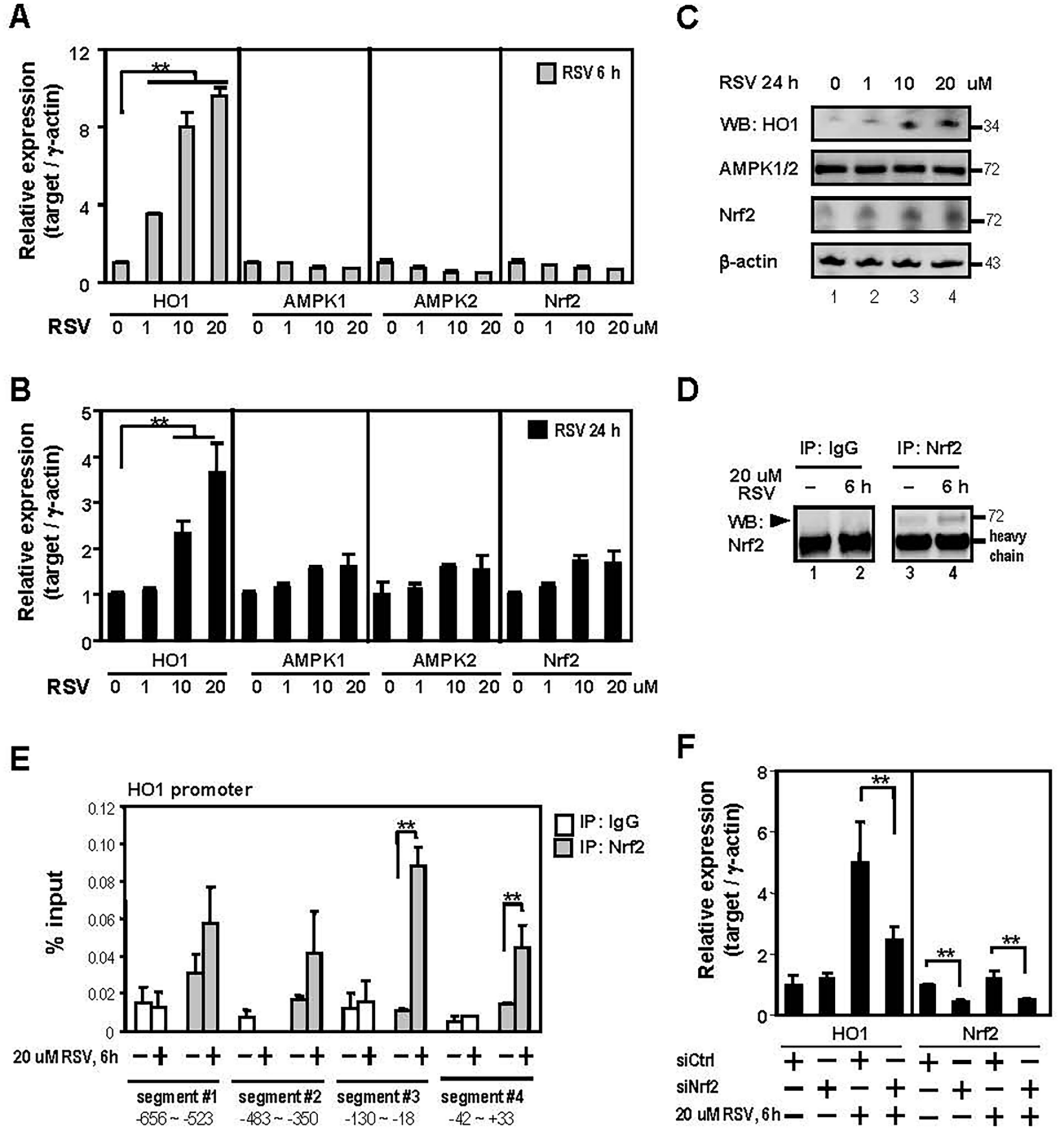
RSV activates HO1 expression via stimulating Nrf2 binding activity in E11 podocytes. (A and B) E11 podocyte cells were treated with or without RSV as indicated. RNA expression levels from RSV-untreated or RSV-treated E11 podocytes were determined by RT-qPCR. Data are presented as the mean ± SD from three independent experiments. **P<0.01. (C) Lysates from RSV-untreated or RSV-treated E11 podocytes were immunoblotted with antibodies against the indicated target. (D) Western blot analysis of immunoprecipitated Nrf2 antibody or an IgG control antibody by anti-Nrf2 antibody from RSV-untreated or RSV-treated E11 podocytes. Arrowhead represents the Nrf2 protein. (E) Chromatin immunoprecipitation (ChIP) assays were performed with antibodies against Nrf2 or an IgG control. Subsequent qPCR analysis was carried out using primers specific for 4 promoter regions of *HO1*. Input represents 1% of the chromatin used for immunoprecipitation. Data are presented as the mean ± SD from three mice. **P<0.01. (F) Real-time qPCR analyses of E11 podocytes transfected with indicated siRNA then treated with 20 um RSV for 6h. Data are presented as the mean ± SD from three independent experiments. **P<0.01.

HO1 levels were increased in glomerular cells from MN-RSV mice compared to control mice (Fig 5E). Given that podocytes play a critical role in the architecture and function of normal glomerulus, we treated E11 podocytes with RSV to independently test whether HO1 would become upregulated at both the mRNA and protein levels. The result showed that concentration-dependent induction of HO1 mRNA by RSV at 6 h and 24 h in E11 podocytes (Fig 7A and 7B). Maximum induction occurred with 20 uM RSV after 6 h (Fig 7A and 7B). Moreover, previous studies indicated that RSV protects against oxidative stress and inflammatory cytokines via AMPK1/2 or Nrf2 signaling[30]. We therefore determined whether the expression of AMPK1/2 and Nrf2 can be regulated by RSV in E11 podocytes. The mRNA expression of AMPK1/2 and Nrf2 did not regulated by RSV at 6 h and 24 h (Fig 7A and 7B). We further examined the protein expression of HO1, AMPK1/2 and Nrf2 in E11 podocytes treated with RSV after 24 h. Consistent with the above-described results, HO1 was highly expressed in cells exposed to RSV at the concentration of 10 and 20 uM RSV (Fig 7C). However, Nrf2 but not AMPK1/2 protein level can be slightly upregulated by RSV (Fig 7C). Similarly, the induction of Nrf2 occurred with 20 uM RSV after 6 h was further confirmed by immunoprecipitation result (Fig 7D). The above results showed that Nrf2 mRNA did not regulated by RSV, we suggesting Nrf2 protein maybe stabilized by RSV in E11 podocyte. Because Nrf2 is a transcription factor capable of forming complex with DNA binding elements, we next wondered whether the elevated Nrf2 by RSV can be reflected on HO1 promoter. Four distinct promoter regions of HO1 were tested, and we found significantly increased Nrf2 in segments 3 and 4 when comparing RSV to no treatment (Fig 7E). Importantly, whether RSV increased Nrf2 recruitment is important for HO1 gene expression. The expression of Nrf2-specific siRNA caused a marked reduction of Nrf2 in E11 podocytes, and the siRNA attenuated the induction of HO1 mRNA by RSV (Fig 7F). Taken together, our data suggests that increased Nrf2 at the HO1 promoter leads to elevated HO1 expression when RSV is exposed.

## Discussion

This is the first study to demonstrate that RSV significantly ameliorates the proteinuria and other pathological characteristics of MN mice. More specifically, the results clearly demonstrate that RSV treatment suppressed complement activation, the oxidative burst, and cell apoptosis in the kidneys of MN mice. RSV also increased the expression of HO1, and inhibition of HO1 by SnPP partially reversed the renoprotective effects of RSV. These results suggest that HO1 may participate in the renoprotective effects of RSV, and that RSV treatment ameliorates experimental MN *via* multiple pathways.

The pathogenesis of MN is characterized by the formation of subepithelial immune deposits, glomerular injury through a complement-dependent processes, and production of oxidants and cytokines, culminating in massive proteinuria [3–5]. Many studies of RSV over the past decade have examined its anticarcinogenic, anti-inflammatory, and estrogenic activities as well as its cardioprotective effects, its scavenging of free-radicals, and its inhibition of apoptosis and platelet aggregation [9, 11, 14, 21]. In our study, RSV treatment effectively attenuated proteinuria by multiple mechanisms, specifically by its immunomodulatory, antioxidative, and antiapoptotic effects. These results suggest that the effect of RSV on systemic inflammation and the concomitant reduction in complement-dependent responses, including oxidative stress, apoptosis, and inflammation, may explain the RSV-mediated attenuation of proteinuria.

The classical complement pathway is central to the pathogenesis of MN. This pathway begins with the formation of antigen-antibody immune complexes, thereby creating a chemoattractant for immune cells, leading to an oxidative burst, increased phagocytosis, release of proteolytic enzymes from intracellular granules, and modulation of cytokine production [31]. However, we found the control and RSV groups had similar immune-complex deposition in the kidneys and similar serum Igs levels. Thus, RSV treatment did not interfere with antibody production, and may have a direct anti-complement activity. Hence, the decreased classical complement activation (including C4, C1 and C3) may be directly due to the anti-complement activity of RSV. A previous study demonstrated that RSV had strong anti-complement activity on the classical complement pathway, but very weak effects on the alternative pathway of the complement system [32, 33]. Furthermore, RSV can also attenuate C5a-induced inflammatory responses by inhibiting the activities of phospholipase D and sphingosine kinase [32]. On the other hand, ROS play a key role in the pathogenesis of MN, and RSV is considered a potent antioxidant and antiapoptotic agent [13, 15]. In fact, RSV treatment dramatically reduced the generation of ROS and proteinuria in our MN mice, suggesting that oxidative stress plays an important role in damaging the glomerular filtration barrier. This may explain the renoprotective effects of RSV. Furthermore, the antiapoptotic effect of RSV based on the observed reduction in TUNEL-positive kidney cells may also contribute to its therapeutic efficacy against MN. Thus, RSV appears to decrease complement activation, reduce oxidative stress and apoptosis, and has direct anti-complement, anti-inflammatory, and anti-oxidative effects, all of which contribute to its renoprotective effects.

The well-known anti-inflammatory activities of RSV may explain its protective effects on numerous conditions, and many studies regard RSV as a potential therapeutic agent for diverse inflammatory diseases [14] [34]. Recent research reported that RSV induced the expression of HO1 [29], a rate-limiting enzyme in the catalysis of heme. There is high expression of RO1 in MN, and RO1 may participate in a critical protective mechanism activated during MN [26]. Previously, we demonstrated that the HO1 induction reduced the severity of MN *via* antioxidative, antiapoptotic, and immunomodulatory mechanisms [26]. The present study indicated that inhibition of HO1 by SnPP attenuated the effect of RSV on MN. Thus HO1 induction is at least partially required for the renoprotective properties of RSV. RSV may target HO1 and thereby induce cellular and organ resistance to kidney damage during MN. We demonstrated here that RSV ameliorates the tissue damage associated with MN by upregulating HO1 expression, an adaptive antioxidative and anti-inflammatory mechanism. Previous studies indicated that RSV protects against oxidative stress and inflammatory cytokines via AMPK1/2 or Nrf2 signaling[30]. Our results is consistent with the above-described results. Furthermore, our data suggests mechanisms that increased Nrf2 at the HO1 promoter leads to elevated HO1 expression when RSV is exposed. Therefore, the HO1 system appears to be one of the most important cytoprotective mechanisms activated during MN, and the protective effects of RSV against MN may partly result from its induction of HO1 expression.

Resveratrol may prevent the development of several clinical conditions, including vascular diseases, cancers, viral infections, and neurodegenerative processes [15]. Additional research indicated that RSV may also be able to treat autoimmune diseases such as type 1 diabetes, rheumatoid arthritis, multiple sclerosis, and inflammatory bowel disease [14]. RSV administration can reduce acute renal lesions in animal models of acute kidney injury, ischemia/reperfusion injury, gentamicin nephrotoxicity, cyclosporin nephrotoxicity, and glycerol-induced renal injury[35]. RSV protects against oxidative stress and inflammatory cytokines *via* AMPK or Nrf2–Keap1 signaling, and this may underlie its beneficial effects on DN [30]. RSV also inhibits proteinuria, hypoalbuminemia, and hyperlipidemia in nephritic rats [36]. We first used RSV in the treatment of nephrotic syndrome in an animal model of autoimmune MN. However, RSV has a short biological half-life, and is metabolized rapidly [37]. Oral administration of 20 mg/kg body weight/day to rats for 28 days led to no treatment-related effects, except for mild changes in serum liver enzymes; a single dose of 2000 mg/kg body weight did not cause any detectable toxicologically significant changes in rats. However, adverse events occurred in rats given 3000 mg/kg body weight/day for 28 days [37]. According to previous studies, even after high-dose RSV administration, only a small amount of the free form is present in plasma. There was a high absorption but very low bioavailability of oral resveratrol in humans. All animal and human studies concur on the poor bioavailability of RSV through low uptake and extensive metabolization[38, 39]. Our study of low-dosage administration of RSV to mice during the early stage of MN indicated that it ameliorated disease severity and did not have any observable adverse effects. Thus, RSV treatment should be considered as an adjuvant therapy for MN.

The development of therapeutic agents that can effectively and specifically obstruct the pathogenesis of MN is an important goal for nephrologists. The currently available immunosuppressive treatments for MN are not always effective and have many adverse effects. In our murine model of MN, RSV blocked the key pathogenic pathways of MN, so an RSV-based therapeutic regimen appears to be a plausible new option for future therapeutic interventions for MN. Clearly, the application of these results to humans requires further research.

In conclusion, we demonstrated that RSV treatment ameliorated MN in a murine model via its anticomplement, antioxidative, antiapoptotic, and immunomodulatory effects. Induction of HO1 may also play a role in the effect of RSV. A treatment regimen that includes RSV should be considered as a potential new therapeutic intervention for MN.
